# Mechanism and Management of Fentanyl-Induced Cough

**DOI:** 10.3389/fphar.2020.584177

**Published:** 2020-10-28

**Authors:** Rong Chen, Ling-hua Tang, Tao Sun, Zi Zeng, Yun-yan Zhang, Ke Ding, Qing-tao Meng

**Affiliations:** ^1^ Department of Anesthesiology, Renmin Hospital of Wuhan University, Wuhan, China; ^2^ Department of Anesthesiology, East Hospital, Renmin Hospital of Wuhan University, Wuhan, China

**Keywords:** adverse drug reactions, opioids, pharmacology, prevention, therapeutics

## Abstract

Fentanyl-induced cough (FIC) often occurs after intravenous bolus administration of fentanyl analogs during induction of general anesthesia and analgesia procedure. The cough is generally benign, but sometimes it causes undesirable side effects, including elevated intra-abdominal, intracranial or intraocular pressure. Therefore, understanding the related mechanisms and influencing factors are of great significance to prevent and treat the cough. This paper reviews the molecular mechanism, influencing factors and preventive administration of FIC, focusing on the efficacy and side effects of various drugs in inhibiting FIC to provide some medical reference for anesthesiologists.

## **Introduction to Fentanyl Analogs**

Opioids, such as fentanyl and its derivatives like sufentanil, remifentanil and alfentanil, are widely used in the induction and maintenance of general anesthesia (Cho et al., 2010; Min et al., 2012). Fentanyl, a µ opioid receptor agonist, was first synthesized in 1960. Its clinical effect is 50–100 times stronger than that of morphine (Stanley, 2014). In some surgical procedures such as laryngoscope and tracheal intubation, it is widely used because of its rapid onset, short acting time, dose-dependent analgesic action, high cardiovascular stability and minimal histamine release (Adachi et al., 2002; Iguchi et al., 2020; Subramaniam et al., 2020).

Like other opioids, fentanyl and its derivatives have a variety of side effects, including nausea, dizziness, constipation, drowsiness, weakness, hypopnea, respiratory depression, etc., which limits their clinical application (Cao et al., 2017; Els et al., 2017; Imam et al., 2018; Wiffen et al., 2014). It has been found that the intravenous administration of fentanyl analogs can cause varying degrees of fentanyl-induced cough (FIC) in some patients (up to 65%) (Gupta et al., 2019; Han et al., 2010). FIC may increase intracranial pressure, intraocular pressure and intra-abdominal pressure, increase the incidence of postoperative nausea and vomiting (Li et al., 2015) and more severely, it may cause a variety of conjunctival and periorbital ecchymosis, and even upper airway obstruction (Gu et al., 2012; Liu et al., 2019). The side effects may be mild and temporary for most patients, but sometimes they may be spastic, explosive or even fatal (Dahan et al., 2010; Lim et al., 2013; Tweed and Dakin, 2001).

Therefore, to explore the mechanism of FIC, to understand the factors affecting the occurrence of cough and to know how to prevent and treat it have an important clinical guidance and physiological significance to reduce the undesirable side effects caused by fentanyl during anesthesia.

## Mechanism of Fentanyl-Induced Cough

The mechanism of FIC is currently not very clear and there are several hypotheses.

### Receptor Hypothesis

Fentanyl analogs can activate µ opioid receptor, and the afferent signal is transmitted to the brainstem through rapidly adapting receptors (stimuli receptors) or vagus nerve C-fiber receptors (J receptors) on the mucosa of the proximal bronchus, subsequently transmitted through the motor fibers in the vagus nerve, causing bronchoconstriction and cough (Böhrer et al., 1990). Fentanyl can significantly increase the number of citric acid-induced coughs in mice and the effect can be antagonized by pretreatment with rapidly adapting receptor antagonist, i.e., moguisteine (Kamei et al., 2013). Pre-inhalation of bronchodilators such as terbutaline or salbutamol can inhibit the bronchoconstriction and cough induced by fentanyl, which also supports this conjecture (Agarwal et al., 2003; Lui et al., 1996).

### Vagal Excitation Hypothesis

Fentanyl analogs may inhibit central sympathetic outflow, and activate vagus nerve activity, causing cough and bronchoconstriction (El Baissari et al., 2014). However, this hypothesis is controversial, some studies have found that pre-administration of anticholinergic drugs such as atropine cannot reduce the incidence of FIC (Lui et al., 1996). While resent study showed that atropine administered in the preoperative period is as effective as lidocaine in preventing FIC (Naldan et al., 2019).

### β-Arrestin Signaling Pathway

**FIGURE 1:**
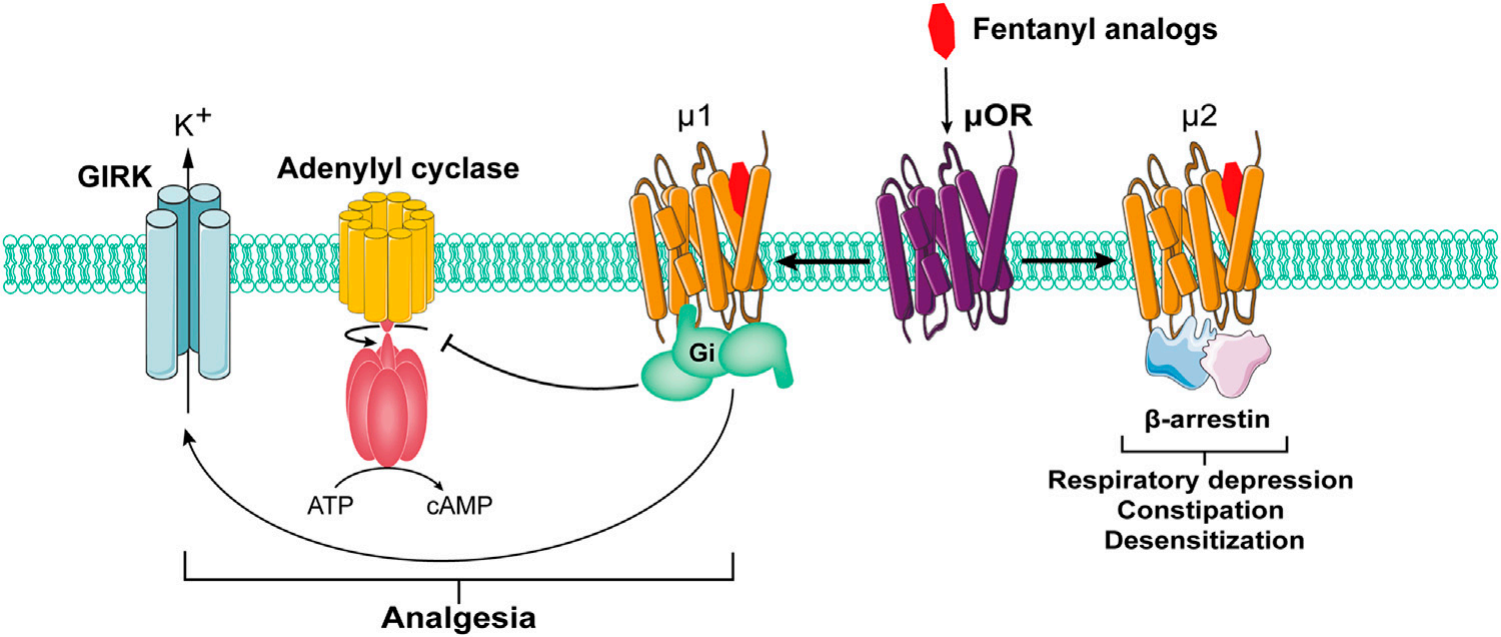
Side effects of opioid receptor agonists mediated by β-arrestin signaling pathway.

As the receptor of opioid drugs, opioid receptor belongs to the G protein-coupled receptor family, which has been confirmed to exist widely in the human nervous system. It has been found that the activation of opioid receptor can activate pertussis toxin-sensitive G protein (Gi and Go) to produce analgesic effects (Lešnik et al., 2020). In this process, the signal pathway mediated by β-arrestin may lead to adverse reactions such as respiratory depression, constipation and desensitization (de Waal et al., 2020) (Figure 1). β-Arrestin knock-out mice exhibit prolonged morphine induced analgesia, increased resistance to respiratory depression, opiate dependence and tolerance, and constipation (Azevedo Neto et al., 2020). However, whether it participates in FIC remains unknown.

### Citric Acid

Tanaka et al. have confirmed that citrate can induce cough in animals and humans by acting on respiratory C fibers (Nurmi et al., 2019; Tanaka and Maruyama, 2005). Fentanyl analogs are mostly prepared in the form of citrate. Therefore, the citric acid contained in those fentanyl analogs stimulates type-C fiber in the smooth muscle of the trachea and bronchi through pulmonary circulation, releasing neuropeptides and causing cough (Böhrer et al., 1990; Kamei et al., 2013). The difference in the incidence and severity of coughs among various opioids may be related to the content of citrate in opioids (El Baissari et al., 2014).

### Opioid Receptor Hypothesis

**FIGURE 2:**
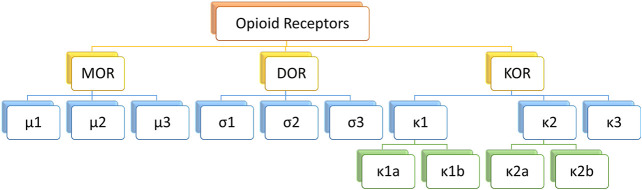
Classification of opioid receptor family.

Classical opioid receptors can be divided into three types: μ, κ and σ. As the most widespread opioid receptors, µ receptors have been confirmed to be widely involved in analgesia and sedation as well as other processes (Li et al., 2020; Valentino and Volkow, 2018) (Figure 2). As µ receptor agonists, fentanyl analogs can bind and activate μ1 receptors and play an analgesic role. However, in the actual medication process, fentanyl analogs can also bind μ2 receptor, causing respiratory depression, nausea and vomiting, cough and other adverse reactions (Raehal et al., 2011).

### Others

Fentanyl analogs may stimulate lung mast cells to release histamine and cause cough. Although fentanyl analogs rarely stimulate histamine release, the application of sodium cromoglycate to inhibit the release of histamine from mast cells can effectively reduce FIC incidence, indicating that histamine may be one of the causes of cough (Kamei et al., 2013). Fentanyl analogs can cause muscle stiffness, resulting in sudden adduction of the vocal cords or soft-tissue obstruction on the glottis, which subsequently induces coughing (Lin et al., 2004). Pre-injection of a low dose of vecuronium to relieve muscle stiffness can reduce FIC incidence (Oshima et al., 2006), indicating that muscle stiffness may also be the cause of cough.

## Influencing Factors of Fentanyl-Induced Cough

Influencing factors of FIC include personal physical condition (gender, age, race, smoking history, disease history, etc.) and fentanyl analogs (type, dose, concentration, injection site, injection rate, etc.) (El Baissari et al., 2014; Shuying et al., 2016).

### Personal Physical Condition

Several studies have demonstrated that the incidence of FIC is higher in infants and young children (Golmohammadi et al., 2018; Han et al., 2010). Young age is one of the important factors for the occurrence of FIC. It has also been found that the Asian population (28%) are more sensitive to fentanyl analogs than the European population (3–6%) (Schäpermeier and Hopf, 2008). The history of smoking is controversial about FIC. Bailey (1999) found that smoking was more likely to induce cough. However, Dimitriou et al. (2006) found that not smoking and heavy smoking (more than 10 cigarettes per day) had no effect on FIC. Lin et al. confirmed the research results of Dimitriou et al. and found that light smoking (<10 cigarettes/day) could prevent FIC (Lin et al., 2005), which may be due to the inhibitory effect of nicotine on type-C fiber in light smokers, but not in heavy smokers (Millqvist and Bende, 2001).

### Evaluation of Fentanyl Analogs

Coughs induced by different fentanyl analogs were variable in occurrence time, incidence and severity. Shen et al. found that equivalent doses of fentanyl, sufentanil and remifentanil had different effects on the incidence, severity and onset time of cough. Remifentanil leads to a higher incidence and severity of cough than the equivalent dose of fentanyl or sufentanil (54.3% vs. 33.3%, 30.5%) (Shen et al., 2008). There was no significant difference in the incidence and severity of cough than the equivalent dose of fentanyl or sufentanil, but the onset time of cough induced by sufentanil was prolonged and its severity was weaker (Shen et al., 2008). An earlier study found that both equivalent doses of sufentanil and fentanyl increased the incidence of cough, but the incidence and severity of cough with sufentanil were weaker than those with fentanyl (Agarwal et al., 2007). The difference between the results of the two studies may be related to the dosage, concentration, route of administration, sample size, race, use of diluent, and drug use by patients prior to the operation.

Many studies have demonstrated that the incidence of cough caused by intravenous administration of fentanyl analogs is proportional to the dose. The incidence of cough will increase from 6.6% to 61% during anesthesia induction with the speed of peripheral intravenous injection of fentanyl less than 2 s, when its dosage is increased from 2 to 4 μg⋅kg^−1^ (El Baissari et al., 2014). Some studies have found that when fentanyl 3 μg⋅kg^−1^ is administered at different drug concentrations (10,25, 50 μg⋅ml^−1^), the incidence of cough induced by 10 μg⋅ml^−1^ fentanyl (12%) is lower than that of 50 μg⋅ml^−1^ (32%) during the same injection time (3–5 s) (Yu et al., 2007).

Cough induced by different routes of fentanyl administration has also been evaluated in many studies. When fentanyl is administered at a high dose via a central vein, the incidence of cough is higher. Bohrer et al. found that the incidence of cough caused by central vein administration of high-dose fentanyl (7 μg⋅kg^−1^) could reach 45.9%, which was significantly higher than 2.7% caused by peripheral administration. This may be due to the direct stimulation of the pulmonary chemical reflex by fentanyl via central vein administration (Böhrer et al., 1990). Chen et al. (2009) found that there was no significant difference in the incidence of cough when the same dose of fentanyl was injected at the same rate through a forearm vein and through the foot or ankle vein, while the occurrence of cough was delayed in the foot or ankle vein group.

Schäpermeier and Hopf (2008) found that the incidence of cough was 3–6% when 1.5 μg⋅kg^−1^ fentanyl was injected at the speed of 2, 5 and 10 s, and they concluded that the injection rate was not related to the incidence of cough. However, Lin et al. (2004) found that further prolongation of the injection time could reduce the incidence of FIC. When 2 μg⋅kg^−1^ fentanyl was injected in under less than 2 s, the incidence of cough was 18%. When the injection time was prolonged to 30 s, the incidence of cough decreased to 1.3% (Lin et al., 2005). Administration of 2 μg⋅kg^−1^ fentanyl via a slow intravenous fluid line at rate of 0.058 µg⋅kg^−1^∙s^−1^ during induction reduced the incidence of FIC than via direct injection 9.1% vs. 55.9%, as were the severity grades (Liu et al., 2017). There were no statistical differences between the two groups with regard to other adverse reactions. When 1.5 μg⋅kg^−1^ remifentanil was injected in pediatric patients, the incidence of FIC decreased from 33% to 5% by increasing the injection time from 30 to 60 s (Kim et al., 2014a). When 4 μg⋅kg^−1^ fentanyl was injected into the peripheral vein (forearm) of the same site at 2 and 15 s, respectively, the incidence of cough was 44% and 8%, and the occurrence time was 16.1 ± 2.7 s and 23.2 ± 3.2 s (Chen et al., 2009). These results indicate that the lower the injection rate is, the lower the incidence and the more delayed the incidence time of cough will be.

## Prevention and Treatment of Fentanyl-Induced Cough

Based on the existing research results, there are numerous pharmacological and non-pharmacological approaches to prevent FIC (Table 1).

**TABLE 1:** Comparison of the effective methods to prevent FIC.

Methods	Details	Limitations
Pharmacological methods		
Fentanyl	A priming dose of 0.5 μg kg^−1^ or 25 μg fentanyl	Nausea and vomiting
Dexmedetomidine	Dexmedetomidine 0.6 μg⋅kg^−1^ given intravenously over 10 min	Bradycardia and hypotension
Ketamine	Ketamine 0.15 mg⋅kg^−1^ given intravenously over 10 s, 1 min before administration of fentanyl	Hallucination and elevated intracranial pressure, intraocular pressure and blood pressure
Propofol	Propofol 10 mg, i.v., 2 min before administration of fentanyl	Injection pain, cardiovascular depression
Lidocaine	Lidocaine 0.5–1.5 mg⋅kg^−1^ given intravenously over 5 s, 1 min before administration of fentanyl	Arrhythmia and depression
Beclomethasone/dexamethasone	Inhaled beclomethasone 15 min before entering the room and fentanyl is given after entering the room Pre-injection of 10 mg dexamethasone	Immunosuppression, metabolic disorder
Dezocine	Dezocine 0.1 mg kg^−1^, i.v., 2 min before administration of sufentanil	Dizzy, nausea and vomiting
Magnesium sulfate (MgSO_4_)	MgSO_4_ 30 mg kg^−1^, i.v., 1 min before administration of fentanyl	50 mg kg^−1^ of MgSO_4_ obvious burning sense, hypermagnesemia
Non-pharmacological methods		
A slow intravenous fluid line	Administration of fentanyl via a slow intravenous fluid line at 0.058 µg⋅kg^−1^∙s^−1^ during induction	Slow, incredible
Mechanical dropper	Administration of fentanyl by mechanical dropper at 1 ml·s^−1^	Slow, incredible
Slow injection of fentanyl	5 µg⋅kg^−1^ fentanyl intravenously injected in 30 s	Slow but effective
Huffing maneuver	Patients were asked to huff (only once) after a deep inspiration. The act of huffing lasted <5 s and was standardized to all patients. Fentanyl injection was started immediately after the completion of huffing maneuver	No limitations, cheap, simple and effective
Swallowing action	Perform the swallowing action immediately before intravenous fentanyl	No limitations, cheap, simple and effective

FIC, fentanyl-induced cough.

### Pharmacological Method

#### The Administration of Fentanyl Analogs

According to the above-mentioned factors related to FIC, the prevention and treatment of FIC can start from the administration of the drug itself, for instance, adjusting the dose and/or concentration of fentanyl analogs, reducing the speed of administration, and selecting appropriate administration routes (such as peripheral administration, etc.). It has also been found that pre-injection of low-dose fentanyl (0.5 μg⋅kg^−1^ or 25 μg directly) can alleviate FIC to some extent (Du et al., 2014; Golmohammadi et al., 2018; Hung et al., 2010; Shrestha et al., 2012). Pretreatment with a small dose of remifentanil 0.3 μg⋅kg^−1^, 1 min before sufentanil injection can effectively and safely reduce the incidence of cough induced by sufentanil (4.8% vs. 31%) (Lin et al., 2019).

#### Bronchodilator

Clonidine, as an α2-adrenoceptor agonist, can inhibit central nervous system excitation. It is known for its analgesic, anti-anxiety and sedative effects, and is widely used in preoperative drug therapy. Horng et al. (2007) found that pre-injection of 2 μg⋅kg^−1^ clonidine 2 min before fentanyl injection could effectively reduce the incidence of cough. The possible mechanism of clonidine in relieving FIC is currently unclear. It has been found that bilateral microinjections of clonidine into the caudal nucleus tractus solitarii (cNTS) or the caudal ventral respiratory group (cVRG) reduce cough responses at 0.5 mmol⋅L^−1^ and abolished the cough reflex at 5 mmol⋅L^−1^ (Cinelli et al., 2013), while oral administration of clonidine does not relieve reflex bronchoconstriction caused by citric acid and capsaicin (O’Connell et al., 1994). Similar to clonidine, pre-injection of dexmedetomidine can effectively reduce the incidence of cough but has no effect on the occurrence time and severity of it (He et al., 2012). He et al. (2012) found that intravenous dexmedetomidine (0.5 or 1 μg⋅kg^−1^) immediately before fentanyl (4 μg⋅kg^−1^) injection reduced the incidence of FIC from 61% to 40%, 18%. Related studies have found that α2-adrenoceptor agonists can reverse muscle stiffness or relieve tracheal smooth muscle contraction caused by histamine, thus reducing the incidence of cough (Mikami et al., 2017). However, as a class of conventional antihypertensive drugs, clonidine and dexmedetomidine are prone to side effects such as bradycardia and hypotension (Shehabi et al., 2019). The risk of bradycardia is significantly higher when the loading dose is greater than 0.7 μg⋅kg^−1^ (Tan and Ho, 2010). Pretreatment with dexmedetomidine intravenous infusion of 0.6 μg⋅kg^−1^ bolus given over 10 min reduced the severity of FIC effectively without adverse effects when fentanyl 4.0 μg⋅kg^−1^ was injected with the injection time of 5 s (Zhou et al., 2019).

Studies have shown that β2-adrenoceptor agonists such as salbutamol, terbutaline, ephedrine, etc. (as bronchial relaxants) can effectively alleviate FIC. Pre-inhalation of salbutamol (concentration not mentioned) or terbutaline (5 mg) 15 min before fentanyl injection can reduce FIC (Agarwal et al., 2007; Lui et al., 1996). Lin et al. (2004) found that the incidence of cough decreased from 65% to 21% in the control group after pre-injection of 5 mg ephedrine.

Kamei et al. (1989) found that *N*-methyl-d-aspartic acid (NMDA) receptors are widely distributed in the larynx, trachea, lung and other tissues, and their agonists can trigger airway contraction and induce cough response. Ketamine is an antagonist of NMDA receptor which can cause bronchiectasis. Pre-injection of 0.15 mg⋅kg^−1^ ketamine 1 min before administration of fentanyl (1.5 μg⋅kg^−1^, injected within 5 s) during induction of general anesthesia can effectively reduce the incidence of cough induced by fentanyl (21.6% vs. 7.2%) and delay its occurrence time 20 s ± 8 s vs. 15 s ± 10 s (Yeh et al., 2007). Durieux (1995) reported that ketamine could effectively block the muscarinic signals and cause bronchiectasis through its peripheral anticholinergic effect. At the same time, Sato et al. (1998) found that ketamine could also inhibit the release of histamine caused by NMDA receptor activation, and then dilate the bronchus. At present, ketamine is limited in use due to its side effects of hallucination and elevated intracranial pressure, intraocular pressure and blood pressure during general anesthesia (Shuying et al., 2016). It has been reported that dextromethorphan, also as an NMDA receptor antagonist, may have similar effects to ketamine (Mukherjee et al., 2011).

Propofol, commonly used in general anesthesia, is found to have tracheal relaxation effects. The mechanism of propofol preventing FIC may be related to the inhibition of NMDA receptor or direct inhibition of bronchoconstriction (Burburan et al., 2007). Pre-emptive use of minimal dose intravenous propofol (20 mg) 1 min prior to 4 μg⋅kg^−1^ fentanyl injection was effective in suppressing a FIC (25.6% vs. 74.4%) (Sedighinejad et al., 2013). Firouzian et al. (2015) found that administration of low dose of propofol (10 mg) 2 min before fentanyl (2 μg⋅kg^−1^) injection over 2 s can reduce the incidence of FIC from 40.8% to 9.2% without accompanying hemodynamic changes. Controversially, Lin et al. (2004) observed that pre-injection of 0.6 mg⋅kg^−1^ propofol could not inhibit FIC.

Lidocaine has been proved to have the effect of reducing airway reflex, including cough response during endotracheal intubation, extubation and bronchography, which may be due to its central role of inhibiting brainstem function, or its peripheral role by blocking the tracheal and pharyngeal cough receptors (Clivio et al., 2019; Sun et al., 2014). Golmohammadi et al. (2018) found that pre-injection of 1 mg⋅kg^−1^ lidocaine could significantly reduce the incidence of FIC. Another study found that 0.5 mg⋅kg^−1^ lidocaine had a significant effect and that increasing the concentration to 1.5 mg⋅kg^−1^ did not further reduce the incidence and severity of FIC (Pandey et al., 2005). A recent meta-analysis also showed that both low (0.5–1 mg⋅kg^−1^) and high doses of lidocaine (1.5–2 mg⋅kg^−1^) were effective at reducing FIC incidence, there was no significant difference between low or high doses of lidocaine (Tan et al., 2018). However, high doses of lidocaine may lead to arrhythmia and depression (Schlimp and Wiedermann, 2005).

#### Antihistamines

Fentanyl analogs may stimulate the release of histamine from lung mast cells to cause cough. Inhibition of histamine release can inhibit FIC. The use of pheniramine maleate (45.5 mg) before induction reduces the incidence of FIC to 2.5% (Ozmen et al., 2016). Beclomethasone is an adrenocortical hormone with anti-inflammatory, anti-allergic and antipruritic effects. It can inhibit bronchial secretion, eliminate the mucosal swelling of bronchus and relieve bronchospasm. It has been confirmed that the incidence of cough decreases to 0 when beclomethasone is inhaled 15 min before entering the room and fentanyl is given at 2 μg⋅kg^−1^ after entering the room, which is significantly lower than that in the control group (28%) (Agarwal et al., 2003). Dexamethasone, as an isomer of beclomethasone, can stabilize mast cells and inhibit histamine release (Lin et al., 2007). At the same time, it can reduce the promoting effect of tachykinin on bronchospasm and stimulate neutral endopeptidase to reverse the increased airway responsiveness of airway epithelial cells (Murlas et al., 1993). Therefore, dexamethasone can be used to prevent FIC. Studies by Yu et al. (2011) and Lin et al. (2007) have both confirmed that pre-injection of 10 mg dexamethasone can effectively reduce the incidence of FIC and cromoglycate can also inhibit histamine release and prevent FIC (Agarwal et al., 2003).

#### Opioid Receptor Agonist and Antagonists

As a κ receptor agonist, dezocine is also a partial µ receptor antagonist. It has been found that pre-injection of dezocine can effectively suppress FIC. Researchers believe that it is possible that dezocine competitively binds to µ receptor, resulting in the decrease of fentanyl binding to µ receptor or inhibition of histamine release, thereby alleviating cough response (Soleimani et al., 2017). A meta-analysis showed that 0.1 mg⋅kg^−1^ dezocine significantly reduced the incidence and severity of sufentanil-induced cough in the induction of general anesthesia, but had no significant effect on vital signs (Xiong et al., 2020). Pentazocine, as an agonist of κ and σ receptors as well as a partial µ receptor antagonist, has been proved to be effective in suppressing cough. According to a study by Qing et al., the incidence of cough can be reduced from 22.6% to 4.3% with pre-injection of 0.5 mg⋅kg^−1^ pentazocine (Ai et al., 2010). In addition, other opioid agonists-antagonists, such as butorphanol, nalbuphine, and oxycodone can also effectively suppress FIC (Cheng et al., 2016; Dai and Cao, 2020; Wang et al., 2020; Yin and Zhang, 2019).

As a selective opioid receptor antagonist, naloxone and nalmefene completely antagonize µ- and δ-opioid receptor and act as a partial agonist at κ-opioid receptor (Ohgi, 2020). A low dose can selectively enhance the analgesic potency of morphine or other opioid agonists, it accounted for by selective antagonism of morphine’s excitatory effects mediated by Gs-coupled opioid receptor functions (Crain and Shen, 2000). Cotreatment with low-dose naloxone also attenuated other hyperexcitability side-effects of morphine, e.g., nausea, vomiting, and pruritus (Gan et al., 1997). Joshi et al. (1999) found that 15 μg nalmefene significantly decreased the need for antiemetics and antipruritic medications in patients receiving intravenous patient-controlled analgesia with morphine. Combined with the mechanism of FIC, related studies have shown that pre-injection of nalmefene can alleviate FIC (Liu et al., 2016; Osborn et al., 2010).

#### Muscle Relaxant

Vecuronium, as a muscle relaxant, has been proved to reduce FIC. The mechanism may be related to a low dose of vecuronium and can effectively relieve muscle stiffness and glottic disorders induced by fentanyl analogs (Horng et al., 2012).

#### Magnesium Sulfate

Magnesium sulfate (MgSO_4_) is reported to have a powerful relaxation of airway smooth muscle, so it is often proposed to treat asthma (Javor and Grle, 2019). Liu et al. found that the incidence of FIC declined from 50.0% to 20.0% and 5.4% after the usage of 30 and 50 mg⋅kg^−1^ of MgSO_4_, respectively. However, because injection of MgSO_4_ can cause hypermagnesemia, which may lead to serious inhibition of neuromuscular excitability, injection with 50 mg⋅kg^−1^ of MgSO_4_ increased the plasma magnesium levels at the end of its infusion and several patients accounting for obvious burning sense (Liu et al., 2015). Pretreatment with MgSO_4_ 30 mg⋅kg^−1^ can safely suppress the incidence and severity of cough induced by fentanyl (5.0 mg⋅kg^−1^) or sufentanil (1.0 μg⋅kg^−1^) during anesthetic induction (An et al., 2015; Liu et al., 2015).

### Non-Pharmacological Methods

Compared to non-pharmacological methods, pharmacological methods may be expensive and may have an added risk of side effects resulting in prolonged hospital stay (Kim et al., 2014b).

Ambesh et al. found that the incidence of cough could be reduced from 32% to 4% without severe FIC in the control group where patients were asked to perform a huffing maneuver prior to the fentanyl injection. The authors speculated that the tracheobronchial stretch receptors might have been pre-treated during that process, hence significantly reduced the incidence of FIC (Ambesh et al., 2010). Gupta et al. (2019) also found that pre-emptive Huff’s maneuver and acupressure could reduce the incidence of fentanyl induced cough. Preoperative incentive spirometry has an important role in anesthesia practice, improving pulmonary function in patients with chronic obstructive and restrictive lung diseases and preventing postoperative atelectasis. Goyal et al. (2017) found that preoperative incentive spirometry before giving fentanyl would suppress FIC, along with the secondary benefit of preventing postoperative pulmonary complications. Furthermore, Sako et al. (2017) showed that swallowing action immediately before intravenous fentanyl also reduce the incidence of FIC from 40.4% to 14.0%.

In summary, in addition to the physiological prevention of FIC, the appropriate drugs for pharmacological treatment of cough include lidocaine, dexmedetomidine, dezocine, low-dose fentanyl and magnesium sulfate (MgSO_4_). Several independent experiments have proved to be effective with relatively minor side effects without affecting the analgesic effect of fentanyl analogs.

## Prospects

Fentanyl analogs are widely used during the perioperative period as commonly used analgesics. However, they are prone to induce FIC which may interfere with the surgical process and postoperative recovery. Therefore, it is necessary to prevent and treat cough during anesthesia. Most of the existing studies are currently focusing on preventing or suppressing FIC, such as a bronchodilator or muscle relaxant. Some achievements have also been made by using agonist antagonists or endogenous antagonists to inhibit the side effects of fentanyl analogs but more clinical samples are needed to verify the dose-response relationship and timing of administration between the above-mentioned drugs and fentanyl analogs. The internal mechanism of FIC is currently unclear and should be the focus of future research, and eventually a safer and more effective preventive treatment method will be found.

## Author Contributions

RC, LT, and TS wrote the manuscript. ZZ, YZ, and KD searched for documents. QM modified the manuscript.

## Conflict of Interest

The authors declare that the research was conducted in the absence of any commercial or financial relationships that could be construed as a potential conflict of interest.

## References

[B1] Adachi Y. U. Satomoto M. Higuchi H. Watanabe K. (2002). Fentanyl attenuates the hemodynamic response to endotracheal intubation more than the response to laryngoscopy. Anesth. Analg. 95, 233–237. 10.1097/00000539-200207000-00043. 12088976

[B2] Agarwal A. Azim A. Ambesh S. Bose N. Dhiraj S. Sahu D. (2003). Salbutamol, beclomethasone or sodium chromoglycate suppress coughing induced by iv fentanyl. Can. J. Anaesth. 50, 297–300. 10.1007/BF03017801 12620955

[B3] Agarwal A. Gautam S. Nath S. S. Gupta D. Singh U. (2007). Comparison of the incidence and severity of cough induced by sufentanil and fentanyl: a prospective, randomised, double-blind study. Anaesthesia 62, 1230–1232. 10.1111/j.1365-2044.2007.05249.x 17991258

[B4] Ai Q. Hu Y. Wang Y. Wu S. Qin Z. Wang J. (2010). Pentazocine pretreatment suppresses fentanyl-induced cough. Pharmacol. Rep. 62, 747–750. 10.1016/s1734-1140(10)70333-9 20885016

[B5] Ambesh S. P. Singh N. Gupta D. Singh P. K. Singh U. (2010). A huffing manoeuvre, immediately before induction of anaesthesia, prevents fentanyl-induced coughing: a prospective, randomized, and controlled study. Br. J. Anaesth. 104, 40–43. 10.1093/bja/aep333 19933512

[B6] An L.-J. Gui B. Su Z. Zhang Y. Liu H.-L. (2015). Magnesium sulfate inhibits sufentanil-induced cough during anesthetic induction. Int. J. Clin. Exp. Med. 8, 13864–13868. 26550339 PMC4613024

[B7] Azevedo Neto J. Costanzini A. De Giorgio R. Lambert D. G. Ruzza C. Calò G. (2020). Biased versus partial agonism in the search for safer opioid analgesics. Molecules 25, 3870 10.3390/molecules25173870 PMC7504468 32854452

[B8] Bailey P. L. (1999). Possible mechanism(s) of opioid-induced coughing. Anesthesiology 90, 335 10.1097/00000542-199901000-00067 9915357

[B9] Böhrer H. Fleischer F. Werning P. (1990). Tussive effect of a fentanyl bolus administered through a central venous catheter. Anaesthesia 45, 18–21. 10.1111/j.1365-2044.1990.tb14496.x 2316832

[B10] Burburan S. M. Xisto D. G. Rocco P. R. M. (2007). Anaesthetic management in asthma. Minerva Anestesiol. 73, 357–365. 17115010

[B11] Cao X. Liu S. Sun J. Yu M. Fang Y. Ding Z. (2017). Fentanyl-induced respiratory depression is attenuated in pregnant patients. Drug Des. Dev. Ther. 11, 3325–3332. 10.2147/DDDT.S147304 PMC5702171 29200828

[B12] Chen Y. Chen W. Liang S. Gu M. (2009). Intravenous injection rate and site of fentanyl affect the incidence and onset time of fentanyl-induced cough. Nan Fang Yi Ke Da Xue Xue Bao 29, 339–340. 19246317

[B13] Cheng X.-Y. Lun X.-Q. Li H.-B. Zhang Z.-J. (2016). Butorphanol suppresses fentanyl-induced cough during general anesthesia induction. Medicine 95, e3911 10.1097/MD.0000000000003911 27367987 PMC4937901

[B14] Cho H. B. Kwak H. J. Park S. Y. Kim J. Y. (2010). Comparison of the incidence and severity of cough after alfentanil and remifentanil injection. Acta Anaesthesiol. Scand. 54, 717–720. 10.1111/j.1399-6576.2009.02203.x 20085544

[B15] Cinelli E. Bongianni F. Pantaleo T. Mutolo D. (2013). Suppression of the cough reflex by α2-adrenergic receptor agonists in the rabbit. Phys. Rep. 1, e00122 10.1002/phy2.122. PMC3871446 24400133

[B16] Clivio S. Putzu A. Tramèr M. R. (2019). Intravenous lidocaine for the prevention of cough: systematic review and meta-analysis of randomized controlled trials. Anesth. Analg. 129, 1249–1255. 10.1213/ANE.0000000000003699 30169416

[B17] Crain S. M. Shen K. F. (2000). Antagonists of excitatory opioid receptor functions enhance morphine’s analgesic potency and attenuate opioid tolerance/dependence liability. Pain 84, 121–131. 10.1016/s0304-3959(99)00223-7 10666516

[B18] Dahan A. Aarts L. Smith T. W. (2010). Incidence, reversal, and prevention of opioid-induced respiratory depression. Anesthesiology 112, 226–238. 10.1097/ALN.0b013e3181c38c25 20010421

[B19] Dai B. Cao X. (2020). Comparing the different oxycodone doses of prevent oxycodone for prevention of preventing fentanyl-induced cough during induction of general anaesthesia. Int. J. Clin. Pract., e13642 10.1111/ijcp.13642 32741071

[B20] de Waal P. W. Shi J. You E. Wang X. Melcher K. Jiang Y. (2020). Molecular mechanisms of fentanyl mediated β-arrestin biased signaling. PLoS Comput. Biol. 16, e1007394 10.1371/journal.pcbi.1007394 32275713 PMC7176292

[B21] Dimitriou V. Spyrou A. Ioakimidou A. Stranomiti J. Koursoumi E. Atsalakis J. (2006). The influence of premedication and smoking. Middle East J. Anesthesiol. 18, 943–946. 17094533

[B22] Du B.-X. Cao L. Zhao W.-L. Xu Z.-H. Song J. Shi X.-Y. (2014). Pre-emptive small dose of fentanyl suppresses fentanyl-induced cough: a meta-analysis of randomized controlled trials. Int. J. Clin. Exp. Med. 7, 826–836. 24955151 PMC4057830

[B23] Durieux M. E. (1995). Inhibition by ketamine of muscarinic acetylcholine receptor function. Anesth. Analg. 81, 57–62. 10.1097/00000539-199507000-00012 7598283

[B24] El Baissari M. C. T. Taha S. K. Siddik-Sayyid S. M. (2014). Fentanyl-induced cough—pathophysiology and prevention. Middle East J. Anesthesiol. 22, 449–456. 25137861

[B25] Els C. Jackson T. D. Kunyk D. Lappi V. G. Sonnenberg B. Hagtvedt R. (2017). Adverse events associated with medium- and long-term use of opioids for chronic non-cancer pain: an overview of Cochrane Reviews. Cochrane Database Syst. Rev. 10, CD012509 10.1002/14651858.CD012509.pub2 29084357 PMC6485910

[B26] Firouzian A. Emadi S. A. Baradari A. G. Mousavi R. Kiasari A. Z. (2015). Can low dose of propofol effectively suppress fentanyl-induced cough during induction of anaesthesia? A double blind randomized controlled trial. J. Anaesthesiol. Clin. Pharmacol. 31, 522–525. 10.4103/0970-9185.169082 26702212 PMC4676244

[B27] Gan T. J. Ginsberg B. Glass P. S. Fortney J. Jhaveri R. Perno R. (1997). Opioid-sparing effects of a low-dose infusion of naloxone in patient-administered morphine sulfate. Anesthesiology 87, 1075–1081. 10.1097/00000542-199711000-00011 9366459

[B28] Golmohammadi M. Shajiee S. Sane S. Valie M. (2018). Comparison of the effects of pretreatment intravenous fentanyl or intravenous lidocaine on suppression of fentanyl-induced cough in children: a randomized, double-blind, controlled clinical trial. Electron. Physician 10, 6877–6883. 10.19082/6877 30034654 PMC6049975

[B29] Goyal V. K. Bhargava S. K. Baj B. (2017). Effect of preoperative incentive spirometry on fentanyl-induced cough: a prospective, randomized, controlled study. Korean J. Anesthesiol. 70, 550–554. 10.4097/kjae.2017.70.5.550 29046775 PMC5645588

[B30] Gu C. Zhou M. Wu H. Li F. Tang Q. (2012). Effects of different priming doses of fentanyl on fentanyl-induced cough: a double-blind, randomized, controlled study. Pharmacol. Rep. 64, 321–325. 10.1016/s1734-1140(12)70771-5 22661182

[B31] Gupta P. Jindal P. Kumar N. (2019). Role of pre-emptive Huff’s manoeuvre and acupressure in reducing the incidence of fentanyl induced cough; a risk factor for postoperative nausea vomiting in female patients: a prospective randomised controlled study. Indian J. Anaesth. 63, 834–840. 10.4103/ija.IJA_549_19 31649396 PMC6798640

[B32] Han J. I. Lee H. Kim C. H. Lee G. Y. (2010). The frequency of fentanyl-induced cough in children and its effects on tracheal intubation. J. Clin. Anesth. 22, 3–6. 10.1016/j.jclinane.2009.01.019 20206844

[B33] He L. Xu J.-M. Dai R.-P. (2012). Dexmedetomidine reduces the incidence of fentanyl-induced cough: a double-blind, randomized, and placebo-controlled study. Ups. J. Med. Sci. 117, 18–21. 10.3109/03009734.2011.629749 22335390 PMC3282237

[B34] Horng H.-C. Lin B.-F. Wang T.-C. Lin S.-L. Liaw W.-J. Wang H.-J. (2012). Priming dose of intravenous rocuronium suppresses fentanyl-induced coughing. Acta Anaesthesiol. Taiwan. 50, 147–149. 10.1016/j.aat.2012.12.003 23385035

[B35] Horng H.-C. Wong C.-S. Hsiao K.-N. Huh B. K. Kuo C.-P. Cherng C.-H. (2007). Pre-medication with intravenous clonidine suppresses fentanyl-induced cough. Acta Anaesthesiol. Scand. 51, 862–865. 10.1111/j.1399-6576.2007.01335.x 17578464

[B36] Hung K.-C. Chen C.-W. Lin V. C.-H. Weng H.-C. Hsieh S.-W. (2010). The effect of pre-emptive use of minimal dose fentanyl on fentanyl-induced coughing. Anaesthesia 65, 4–7. 10.1111/j.1365-2044.2009.06109.x 19889113

[B37] Iguchi N. Kosaka J. Iguchi Y. Evans R. G. Bellomo R. May C. N. (2020). Systemic haemodynamic, renal perfusion and renal oxygenation responses to changes in inspired oxygen fraction during total intravenous or volatile anaesthesia. Br. J. Anaesth. 125, 192–200. 10.1016/j.bja.2020.03.033 32563492

[B38] Imam M. Z. Kuo A. Ghassabian S. Smith M. T. (2018). Progress in understanding mechanisms of opioid-induced gastrointestinal adverse effects and respiratory depression. Neuropharmacology 131, 238–255. 10.1016/j.neuropharm.2017.12.032 29273520

[B39] Javor E. Grle S. P. (2019). Limitations of the results from randomized clinical trials involving intravenous and nebulised magnesium sulphate in adults with severe acute asthma. Pulm. Pharmacol. Ther. 55, 31–37. 10.1016/j.pupt.2019.01.005 30660759

[B40] Joshi G. P. Duffy L. Chehade J. Wesevich J. Gajraj N. Johnson E. R. (1999). Effects of prophylactic nalmefene on the incidence of morphine-related side effects in patients receiving intravenous patient-controlled analgesia. Anesthesiology 90, 1007–1011. 10.1097/00000542-199904000-00013 10201671

[B41] Kamei J. Nakanishi Y. Asato M. Ikeda H. (2013). Fentanyl enhances the excitability of rapidly adapting receptors to cause cough via the enhancement of histamine release in the airways. Cough 9, 3 10.1186/1745-9974-9-3 23369146 PMC3566957

[B42] Kamei J. Tanihara H. Igarashi H. Kasuya Y. (1989). Effects of N-methyl-D-aspartate antagonists on the cough reflex. Eur. J. Pharmacol. 168, 153–158. 10.1016/0014-2999(89)90560-8 2691260

[B43] Kim D.-H. Yoo J.-Y. Moon B.-K. Yoon B.-H. Kim J.-Y. (2014a). The effect of injection speed on remifentanil-induced cough in children. Korean J. Anesthesiol. 67, 171–174. 10.4097/kjae.2014.67.3.171 25301090 PMC4188761

[B44] Kim J. E. Min S. K. Chae Y. J. Lee Y. J. Moon B. K. Kim J. Y. (2014b). Pharmacological and nonpharmacological prevention of fentanyl-induced cough: a meta-analysis. J. Anesth. 28, 257–266. 10.1007/s00540-013-1695-4 23958914

[B45] Lešnik S. Hodošček M. Bren U. Stein C. Bondar A.-N. (2020). Potential energy function for fentanyl-based opioid pain killers. J. Chem. Inf. Model. 60, 3566–3576. 10.1021/acs.jcim.0c00185 32491854

[B46] Li C. C. Chen S. S. Huang C. H. Chien K. L. Yang H. J. Fan S. Z. (2015). Fentanyl-induced cough is a risk factor for postoperative nausea and vomiting. Br. J. Anaesth. 115, 444–448. 10.1093/bja/aev157 26034022

[B47] Li G. Nieman A. N. Mian M. Y. Zahn N. M. Mikulsky B. N. Poe M. M. (2020). A structure-activity relationship comparison of imidazodiazepines binding at kappa, mu, and delta opioid receptors and the GABAA receptor. Molecules 25, 3864 10.3390/molecules25173864 PMC7503500 32854311

[B48] Lim K. J. Lee S. K. Lee H. M. Park E. Y. Kim M. H. Kim Y. S. (2013). Aspiration pneumonia caused by fentanyl-induced cough—a case report. Korean J. Anesthesiol. 65, 251–253. 10.4097/kjae.2013.65.3.251 24101960 PMC3790037

[B49] Lin C.-S. Sun W.-Z. Chan W.-H. Lin C.-J. Yeh H.-M. Mok M. S. (2004). Intravenous lidocaine and ephedrine, but not propofol, suppress fentanyl-induced cough. Can. J. Anaesth. 51, 654–659. 10.1007/BF03018421 15310631

[B50] Lin J.-A. Chen F.-C. Lee M.-S. Horng H.-C. Cherng C.-H. Yeh C.-C. (2007). Intravenous dexamethasone pretreatment reduces fentanyl-induced cough. J. Formos. Med. Assoc. 106, 649–655. 10.1016/S0929-6646(08)60022-4 17711798

[B51] Lin J.-A. Yeh C.-C. Lee M.-S. Wu C.-T. Lin S.-L. Wong C.-S. (2005). Prolonged injection time and light smoking decrease the incidence of fentanyl-induced cough. Anesth. Analg. 101, 670–674. 10.1213/01.ane.0000159161.31276.db 16115973

[B52] Lin W. Sun J. Fu S. (2019). A small dose of remifentanil pretreatment suppresses sufentanil-induced cough during general anesthesia induction: a randomized, double-blind, placebo-controlled trial. BMC Anesthesiol. 19, 164 10.1186/s12871-019-0836-1 31455295 PMC6712682

[B53] Liu C. Liu M. Zhang Z. (2016). Efficacy of nalmefene in preventing sufentanil-induced cough during induction of general anesthesia. Chin. J. Anesthesiol. 36, 1441–1443.

[B54] Liu H.-L. An L.-J. Su Z. Zhang Y. Gui B. (2015). Magnesium sulphate suppresses fentanyl-induced cough during general anesthesia induction: a double-blind, randomized, and placebo-controlled study. Int. J. Clin. Exp. Med. 8, 11332–11336. 26379945 PMC4565328

[B55] Liu M. Li F. Han Y. He J. Shi H. Liu L. (2017). Administration of fentanyl via a slow intravenous fluid line compared with rapid bolus alleviates fentanyl-induced cough during general anesthesia induction. J. Zhejiang Univ. Sci. B 18, 955–962. 10.1631/jzus.B1600442 29119733 PMC5696314

[B56] Liu M. Li Z. Wang S. Liu Y. Zhong X. He R. (2019). Application via mechanical dropper alleviates sufentanil-induced cough: a prospective, randomized, single-blinded trial. Trials 20, 170 10.1186/s13063-019-3274-y 30876430 PMC6420750

[B57] Lui P. W. Hsing C. H. Chu Y. C. (1996). Terbutaline inhalation suppresses fentanyl-induced coughing. Can. J. Anaesth. 43, 1216–1219. 10.1007/BF03013427 8955969

[B58] Mikami M. Zhang Y. Kim B. Worgall T. S. Groeben H. Emala C. W. (2017). Dexmedetomidine’s inhibitory effects on acetylcholine release from cholinergic nerves in Guinea pig trachea: a mechanism that accounts for its clinical benefit during airway irritation. BMC Anesthesiol. 17, 52 10.1186/s12871-017-0345-z 28356076 PMC5372301

[B59] Millqvist E. Bende M. (2001). Capsaicin cough sensitivity is decreased in smokers. Respir. Med. 95, 19–21. 10.1053/rmed.2000.0965 11207012

[B60] Min S. K. Kim D. H. Cho H. B. Moon B. K. Kim J. Y. (2012). Limited maximal flow rate of target-controlled remifentanil infusion and induced cough. Anaesthesia 67, 145–148. 10.1111/j.1365-2044.2011.06961.x 22251106

[B61] Mukherjee A. Kundu A. K. Ghosh S. Choudhuri R. Bandopadhyay B. K. Dasgupta S. (2011). Pre-emptive oral dexmethorphan reduces fentanyl-induced cough as well as immediate postoperative adrenocortico-tropic hormone and growth hormone level. J. Anaesthesiol. Clin. Pharmacol. 27, 489–494. 10.4103/0970-9185.86593 22096282 PMC3214554

[B62] Murlas C. G. Lang Z. Chodimella V. (1993). Dexamethasone reduces tachykinin but not ACh airway hyperreactivity after O3. Lung 171, 109–121. 10.1007/BF00542338 7678875

[B63] Naldan M. E. Arslan Z. Ay A. Yayık A. M. (2019). Comparison of lidocaine and atropine on fentanyl-induced cough: a randomized controlled study. J. Invest. Surg. 32, 428–432. 10.1080/08941939.2018.1424272 29388856

[B64] Nurmi H. M. Lätti A. M. Brannan J. D. Koskela H. O. (2019). Comparison of mannitol and citric acid cough provocation tests. Respir. Med. 158, 14–20. 10.1016/j.rmed.2019.09.011 31542680

[B65] O’Connell F. Thomas V. E. Fuller R. W. Pride N. B. Karlsson J. A. (1994). Effect of clonidine on induced cough and bronchoconstriction in guinea pigs and healthy humans. J. Appl. Physiol. 76, 1082–1087. 10.1152/jappl.1994.76.3.1082 8005849

[B66] Ohgi Y. (2020). Alcohol dependence and opioid receptor—pharmacological profile of nalmefene. Nihon Yakurigaku Zasshi 155, 145–148. 10.1254/fpj.19139 32378631

[B67] Osborn M. D. Lowery J. J. Skorput A. G. J. Giuvelis D. Bilsky E. J. (2010). *In vivo* characterization of the opioid antagonist nalmefene in mice. Life Sci. 86, 624–630. 10.1016/j.lfs.2010.02.013 20159022 PMC2848904

[B68] Oshima T. Kasuya Y. Okumura Y. Murakami T. Dohi S. (2006). Identification of independent risk factors for fentanyl-induced cough. Can. J. Anaesth. 53, 753–758. 10.1007/BF03022790 16873340

[B69] Ozmen O. Kara D. Karaman E. U. Karakoc F. Karakaya M. A. Arslan Z. (2016). Pheniramine maleate is more effective than lidocaine on fentanyl induced cough. Pak. J. Med. Sci. 32, 715–719. 10.12669/pjms.323.9496 27375720 PMC4928429

[B70] Pandey C. K. Raza M. Ranjan R. Singhal V. Kumar M. Lakra A. (2005). Intravenous lidocaine 0.5 mg.kg^-1^ effectively suppresses fentanyl-induced cough. Can. J. Anaesth. 52, 172–175. 10.1007/BF03027724 15684258

[B71] Raehal K. M. Schmid C. L. Groer C. E. Bohn L. M. (2011). Functional selectivity at the μ-opioid receptor: implications for understanding opioid analgesia and tolerance. Pharmacol. Rev. 63, 1001–1019. 10.1124/pr.111.004598 21873412 PMC3186080

[B72] Sako S. Tokunaga S. Tsukamoto M. Yoshino J. Fujimura N. Yokoyama T. (2017). Swallowing action immediately before intravenous fentanyl at induction of anesthesia prevents fentanyl-induced coughing: a randomized controlled study. J. Anesth. 31, 212–218. 10.1007/s00540-016-2300-4 28050704

[B73] Sato T. Hirota K. Matsuki A. Zsigmond E. K. Rabito S. F. (1998). The role of the N-methyl-D-aspartic acid receptor in the relaxant effect of ketamine on tracheal smooth muscle. Anesth. Analg. 87, 1383–1388. 10.1097/00000539-199812000-00033 9842833

[B74] Schäpermeier U. Hopf H.-B. (2008). Fentanyl-induced cough does not depend on injection speed: a randomized study. Acta Anaesthesiol. Scand. 52, 1071–1075. 10.1111/j.1399-6576.2008.01721.x 18840106

[B75] Schlimp C. J. Wiedermann F. J. (2005). Does fentanyl-induced cough justify pre-treatment with iv lidocaine 2 mg.kg^-1^ . Can. J. Anaesth. 52, 207 10.1007/BF03027731 15684266

[B76] Sedighinejad A. Naderi Nabi B. Haghighi M. Imantalab V. Hadadi S. Erfani Sayar R. (2013). Propofol is effective to depress fentanyl-induced cough during induction of anesthesia. Anesth. Pain Med. 2, 170–173. 10.5812/aapm.8383 24223355 PMC3821139

[B77] Shehabi Y. Howe B. D. Bellomo R. Arabi Y. M. Bailey M. Bass F. E. (2019). Early sedation with dexmedetomidine in critically ill patients. N. Engl. J. Med. 380 (26), 2506–2517. 10.1056/NEJMoa1904710 31112380

[B78] Shen J.-C. Xu J.-G. Zhou Z.-Q. Liu H.-J. Yang J.-J. (2008). Effect of equivalent doses of fentanyl, sufentanil, and remifentanil on the incidence and severity of cough in patients undergoing abdominal surgery: a prospective, randomized, double-blind study. Curr. Ther. Res. Clin. Exp. 69, 480–487. 10.1016/j.curtheres.2008.12.002 24692822 PMC3969975

[B79] Shrestha S. K. Bhattarai B. Shah R. S. (2012). Preemptive use of small dose fentanyl suppresses fentanyl induced cough. Kathmandu Univ. Med. J. 10, 16–19. 10.3126/kumj.v10i4.10988 23575046

[B80] Shuying L. Ping L. Juan N. Dong L. (2016). Different interventions in preventing opioid-induced cough: a meta-analysis. J. Clin. Anesth. 34, 440–447. 10.1016/j.jclinane.2016.05.034 27687431

[B81] Soleimani A. Kiabi F. H. Habibi M. R. Emami Zeydi A. Assarroudi A. Sharifi H. (2017). Intravenous dezocine for suppressing fentanyl-induced cough during general anesthesia induction: a potentially effective and clinically feasible method. J. Anaesthesiol. Clin. Pharmacol. 33, 556–557. 10.4103/0970-9185.222514 29416260 PMC5791281

[B82] Stanley T. H. (2014). The fentanyl story. J. Pain 15, 1215–1226. 10.1016/j.jpain.2014.08.010 25441689

[B83] Subramaniam K. Sciortino C. Ruppert K. Monroe A. Esper S. Boisen M. (2020). Remifentanil and perioperative glycaemic response in cardiac surgery: an open-label randomised trial. Br. J. Anaesth. 124, 684–692. 10.1016/j.bja.2020.01.028 32247539

[B84] Sun L. Guo R. Sun L. (2014). The impact of prophylactic intravenous lidocaine on opioid-induced cough: a meta-analysis of randomized controlled trials. J. Anesth. 28, 325–333. 10.1007/s00540-013-1732-3 24173406

[B85] Tan J. A. Ho K. M. (2010). Use of dexmedetomidine as a sedative and analgesic agent in critically ill adult patients: a meta-analysis. Intensive Care Med. 36, 926–939. 10.1007/s00134-010-1877-6 20376429

[B86] Tan W. Li S. Liu X. Gao X. Huang W. Guo J. (2018). Prophylactic intravenous lidocaine at different doses for fentanyl-induced cough (FIC): a meta-analysis. Sci. Rep. 8, 9946 10.1038/s41598-018-27457-3 29967371 PMC6028622

[B87] Tanaka M. Maruyama K. (2005). Mechanisms of capsaicin- and citric-acid-induced cough reflexes in guinea pigs. J. Pharmacol. Sci. 99, 77–82. 10.1254/jphs.fpj05014x 16127241

[B88] Tweed W. A. Dakin D. (2001). Explosive coughing after bolus fentanyl injection. Anesth. Analg. 92, 1442–1443. 10.1097/00000539-200106000-00018 11375822

[B89] Valentino R. J. Volkow N. D. (2018). Untangling the complexity of opioid receptor function. Neuropsychopharmacology 43, 2514–2520. 10.1038/s41386-018-0225-3 30250308 PMC6224460

[B90] Wang J. Duan J. Wang Q. Lu Y. (2020). Pretreatment with nalbuphine prevents sufentanil-induced cough during the anesthesia induction: a randomized controlled trial. Ther. Clin. Risk Manage. 16, 281–286. 10.2147/TCRM.S247437 PMC7166053 32341646

[B91] Wiffen P. J. Derry S. Moore R. A. (2014). Impact of morphine, fentanyl, oxycodone or codeine on patient consciousness, appetite and thirst when used to treat cancer pain. Cochrane Database Syst. Rev. 2014, CD011056 10.1002/14651858.CD011056.pub2 PMC6483540 24874470

[B92] Xiong Z. Yi P. Song J. Tan M. (2020). Dezocine prevents sufentanil-induced cough during general anesthesia induction: a meta-analysis of randomised controlled trials. BMC Anesthesiol. 20, 154 10.1186/s12871-020-01076-w 32571219 PMC7310133

[B93] Yeh C.-C. Wu C.-T. Huh B. K. Lee M.-S. Lin S.-L. J Sheen M. (2007). Premedication with intravenous low-dose ketamine suppresses fentanyl-induced cough. J. Clin. Anesth. 19, 53–56. 10.1016/j.jclinane.2006.05.021 17321928

[B94] Yin F. Zhang T. (2019). A small dose of butorphanol prevents sufentanil-induced cough during general anesthesia induction. J. Craniofac. Surg. 30, 2499–2501. 10.1097/SCS.0000000000005967 31567764

[B95] Yu H. Yang X.-Y. Zhang X. Li Q. Zhu T. Wang Y. (2007). The effect of dilution and prolonged injection time on fentanyl-induced coughing. Anaesthesia 62, 919–922. 10.1111/j.1365-2044.2007.05147.x 17697219

[B96] Yu M.-S. Kim J. Y. Kim H. Y. (2011). Intravenous dexamethasone pretreatment reduces remifentanil induced cough. Korean J. Anesthesiol. 60, 403–407. 10.4097/kjae.2011.60.6.403 21738842 PMC3121086

[B97] Zhou W. Zhang D. Tian S. Yang Y. Xing Z. Ma R. (2019). Optimal dose of pretreated-dexmedetomidine in fentanyl-induced cough suppression: a prospective randomized controlled trial. BMC Anesthesiol. 19, 89 10.1186/s12871-019-0765-z 31153360 PMC6545214

